# Registry of the Spanish Network for Systemic Sclerosis: Survival, Prognostic Factors, and Causes of Death

**DOI:** 10.1097/MD.0000000000001728

**Published:** 2015-10-30

**Authors:** C.P. Simeón-Aznar, V. Fonollosa-Plá, Carles Tolosa-Vilella, G. Espinosa-Garriga, M. Campillo-Grau, M. Ramos-Casals, F.J. García-Hernández, M.J. Castillo-Palma, J. Sánchez-Román, J.L. Callejas-Rubio, N. Ortego-Centeno, M.V. Egurbide-Arberas, L. Trapiellla-Martínez, L. Caminal-Montero, L. Sáez-Comet, J. Velilla-Marco, M.T. Camps-García, E. de Ramón-Garrido, E.M. Esteban-Marcos, L. Pallarés-Ferreres, N. Navarrete-Navarrete, J.A. Vargas-Hitos, R. Gómez de la Torre, G. Salvador-Cervello, J.J. Rios-Blanco, M. Vilardell-Tarrés

**Affiliations:** From the Department of Internal Medicine, Hospital Valld’Hebron (CPS-A, VF-P, MV-T); Department of Internal Medicine, Hospital Parc Taulí, Sabadell (CT-V); Department of Autoimmune Diseases, Hospital Clinic (GE-G, MR-C); Laboratori of Computacional Medicine, Bioestatistics Unit, Universitat Autònoma de Barcelona, Bellaterra, Barcelona (MC-G); Unit of Connective Tissue Diseases, Department of Internal Medicine, Hospital Virgen del Rocio, Sevilla (FJG-H, MJC-P, JS-R); Unit of Autoimmune Systemic Diseases, Department of Internal Medicine, Hospital Clínico San Cecilio, Granada (JLC-R, NO-C); Department of Internal Medicine, Hospital de Cruces, Galdakano, Bilbao (MVE-A); Department of Internal Medicine, Hospital de Cabueñes, Gijón (LT-M); Department of Internal Medicine, Hospital Universitario Central de Asturias, Oviedo (LC-M); Department of Internal Medicine, Hospital Miguel Servet, Zaragoza (LS-C, JV-M); Department of Internal Medicine, Hospital Carlos Haya, Málaga (MTC-G, ER-G); Department of Internal Medicine, Hospital Son Espases, Palma de Mallorca (EME-M, LP-F); Department of Internal Medicine, Hospital Virgen de las Nieves, Granada (NN-N, JAV-H); Department of Internal Medicine, Hospital San Agustín, Avilés (RGT); Department of Internal Medicine, Hospital La Fe, Valencia (GS-C); and Department of Internal Medicine, Hospital La Paz, Madrid (JJR-B), Systemic Autoimmune Diseases Group (GEAS), Spanish Scleroderma Study Group (SSSG), Spanish Society of Internal Medicine, Spain.

## Abstract

Systemic sclerosis (SSc) is a rare, multisystem disease showing a large individual variability in disease progression and prognosis. In the present study, we assess survival, causes of death, and risk factors of mortality in a large series of Spanish SSc patients. Consecutive SSc patients fulfilling criteria of the classification by LeRoy were recruited in the survey. Kaplan–Meier and Cox proportional-hazards models were used to analyze survival and to identify predictors of mortality. Among 879 consecutive patients, 138 (15.7%) deaths were registered. Seventy-six out of 138 (55%) deceased patients were due to causes attributed to SSc, and pulmonary hypertension (PH) was the leading cause in 23 (16.6%) patients. Survival rates were 96%, 93%, 83%, and 73% at 5, 10, 20, and 30 years after the first symptom, respectively. Survival rates for diffuse cutaneous SSc (dcSSc) and limited cutaneous SSc were 91%, 86%, 64%, and 39%; and 97%, 95%, 85%, and 81% at 5, 10, 20, and 30 years, respectively (log-rank: 67.63, *P* < 0.0001). The dcSSc subset, male sex, age at disease onset older than 65 years, digital ulcers, interstitial lung disease (ILD), PH, heart involvement, scleroderma renal crisis (SRC), presence of antitopoisomerase I and absence of anticentromere antibodies, and active capillaroscopic pattern showed reduced survival rate. In a multivariate analysis, older age at disease onset, dcSSc, ILD, PH, and SRC were independent risk factors for mortality. In the present study involving a large cohort of SSc patients, a high prevalence of disease-related causes of death was demonstrated. Older age at disease onset, dcSSc, ILD, PH, and SRC were identified as independent prognostic factors.

## INTRODUCTION

Systemic sclerosis (SSc) is a rare, multisystem disease showing a large individual variability in the extent of skin and organ involvement, as well as in disease progression and prognosis. Its natural course varies from a relatively benign condition to a rapidly progressive disease, with a high risk of mortality.^1^ Many studies have evaluated survival and predictors of poor outcome in patients with SSc.^2–8^ However, this disease is relatively uncommon, and results from small series of patients may be nonrepresentative. In this sense, nationwide registers are required to identify prognostic factors and the natural history of SSc in real life.^9–13^ Large registries are important to define a more uniform cohort of patients that allow recruiting patients into clinical studies with comparable criteria for outcome measurements. Up to now, only few multicentric studies examining survival in SSc patients have been reported with considerable differences among them.^3,14–16^ Main explanations to these controversies are differences on inclusion criteria and differences on definition of clinical manifestations, survival estimation from different time points (from disease onset or from diagnosis of SSc), heterogeneity of disease course, and different proportion of patients with diffuse and limited SSc included in the series.

The extent of skin sclerosis has been considered an important prognostic factor by several authors.^5,17–25^ In this sense, SSc patients are usually subdivided into diffuse cutaneous (dcSSc) and limited cutaneous (lcSSc) subsets according to the most worldwide used classification proposed by LeRoy et al.^26^ However, a third subset of patients named SSc sine scleroderma (ssSSc) and defined by the presence of Raynaud phenomenon (RP), any typical scleroderma visceral involvement and positive antinuclear antibodies, but no cutaneous sclerosis, was taken into account. This SSc subset has not usually been considered in survival studies even though different clinical characteristics between ssSSc and lcSSc have been found by some authors.^27,28^ In our previous study about clinical pattern according to cutaneous subsets and immunological status of SSc patients, ssSSc subset was already considered, and we detected a significant higher prevalence of pulmonary and cardiac involvement in ssSSc patients than in lcSSc subset.^29^

The importance of skin sclerosis and visceral involvement as prognostic factors in SSc patients is not always concordant among different studies.^2–8,19–24^ Moreover, survival heterogeneity was already evidenced by Pope^30^ in an Editorial that included two meta-analyses about survival and mortality.^31,32^ Recently, causes and risk factors for death in SSc were also reported by the The European League Against Rheumatism (EULAR). In a multinational database by the EULAR Scleroderma Trials and Research (EUSTAR), 55% of deaths were directly related to SSc, to lead by interstitial lung disease (ILD), pulmonary arterial hypertension (PAH), and cardiac involvement.^25^ The independent mortality risk factors in that series were the presence of proteinuria on the urine dipstick analysis, dyspnea on exertion, PAH, pulmonary restriction with lesser than 80% of the predicted forced vital capacity (FVC), low diffusing capacity of the lung for carbon monoxide (DLCO), older age at onset of RP, and a modified Rodnan skin score (mRSS) higher than 10. Intriguingly, SSc cutaneous subtype, autoantibody status, and sex were not independent predictors of subsequent death.

The present survey is a retrospective study of a cohort of SSc patients enrolled in the first Spanish nationwide registry by the Spanish Scleroderma Study Group (SSSG) or RESCLE (*Registro de ESCLErodermia* as Spanish nomenclature) Registry. The aim of this study was to analyze survival from disease onset, causes of death, and risk factors associated with mortality in a large series of SSc patients.

## METHODS

### Study Cohort

The SSSG or RESCLE was created by the Spanish Internal Medicine Society in 2006, with the aim of compiling a large series of Spanish patients with SSc. Fourteen Spanish centers with experience in the management of these patients participated in the patient recruitment. All participating centers had obtained ethics committee approval. To avoid excluding patients with clear diagnosis who did not fulfill the American College of Rheumatology (ACR) preliminary classification criteria for SSc,^33^ we used a modification of the classification proposed by LeRoy and Medsger.^34^ Thus, we considered ssSSc patients as an independent subset of lcSSc to extend the previously reported results from the same cohort of patients.^29^ Patients with SSc registered as deceased up to January 2008 were also analyzed. Epidemiological (including time and cause of death), clinical, laboratory, capillarocopic, and immunological data encompassing 90 variables were recorded according to a standard protocol designed by the SSSG,^5,6^ and were entered into a SPSS database. Disease onset was defined as the date of the first self-reported symptom (RP in the majority of patients) following the criteria of our previous study and in agreement with other studies.^1,5,6,15,16,25,35–41^

### Cutaneous Subsets

Three groups of SSc patients were established according to the extent of skin sclerosis, following a modification of the classification proposed by LeRoy and Medsger:^34^

1. *lcSSc*, when the skin sclerosis was confined distally to the elbows and knees and/or the face,
2. *dcSSc*, when the skin thickening extended proximally to the elbows and knees or included the trunk, and
3. *ssSSc*, defined by the presence of RP or a peripheral vascular equivalent (digital pitting scars, fingertip ulcers, SSc-type nailfold capillary pattern), scleroderma clinical features (gastrointestinal hypomotility, ILD, PAH, typical cardiac involvement, or scleroderma renal crisis [SRC]), and positive antinuclear antibodies, but no skin sclerosis.

### Clinical Characteristics

“Peripheral vascular manifestations” are defined by the presence of RP, ischemic digital ulcerations, and acro-osteolysis (tuft resorption secondary to digital ischemia identified in the radiological study by radiologist). “Digestive tract involvement” considers any of the following diagnoses related to SSc: *esophageal involvement*, when hypomotility of the lower two-thirds of the esophagus and/or decreased peristalsis were confirmed by manometry or cine-radiographic study; *gastric involvement*, when gastric hypomotility was detected by radiographic or radionuclide study, or when gastric antral vascular ectasia was identified by endoscopy; *intestinal involvement*, when an intestinal motility disturbance was confirmed by manometry or cine-radiographic study, or malabsorption syndrome was diagnosed by breath test, or intestinal pseudo-obstruction was identified by simple radiology or computerized tomography scan^5,6^; *hepatic involvement*, when diagnoses of primary biliary cirrhosis, autoimmune hepatitis, or nodular regenerative hyperplasia of the liver were established. “Pulmonary involvement” is defined by the presence of ILD or pulmonary hypertension (PH). ILD was established if any of the following criteria were identified: restrictive pulmonary pattern with FVC below 80% of expected value on pulmonary function tests without other causes, pulmonary interstitial pattern evidenced by chest radiograph or high-resolution computed tomography (HRCT) scan, or alveolitis confirmed by bronchoalveolar lavage (defined as neutrophilia of ≥3%, eosinophilia of ≥2%, or lymphocytosis ≥15%). PH was considered when systolic pulmonary arterial pressure was estimated to be above 40 mm Hg by Doppler echocardiogram corresponding to maximum tricuspid regurgitant jet velocity of 3.0 m/s to 3.5 m/s, or when mean pulmonary arterial pressure (PAP) was found to be higher or equal to 25 mm Hg by right-sided heart catheterization (RSHC).^5,6^ PH was considered to be isolated when ILD was not identified or was mild (FVC >70%). PH was classified as ILD-associated if the patient had a restrictive ventilator pattern with FVC <70% and severe lung fibrosis in HRCT. “Muscle involvement” is defined by the presence of proximal muscle weakness or myalgias and at least one of the following abnormalities: serum creatine kinase (CK) over the normal value or results of an electromyography (EMG) consistent with myopathy. “Joint and tendon involvement” is defined by the presence of any of the following: arthralgia, arthritis, and tendon friction rubs. “Heart involvement” was established by one or more of the following: pericarditis, ischemic cardiomyopathy with no known cause, reversible thallium perfusion defects after cold stimulation,^42^ any disturbance on color-Doppler echocardiography, electrocardiographic alterations with no other cause, left ventricular ejection fraction lower than 50% or right ventricular ejection fraction lower than 40% on echocardiography or radionuclide ventriculography. We assumed that ischemic cardiomyopathy was SSc-related only in the absence of other vascular risk factors. “Scleroderma renal crisis” is defined by the presence of a rapid deterioration of renal function (with concomitant normal urine sediment) within a period of less than 1 month in the absence of previous evidence of significant kidney disease or by the combination of abrupt onset or aggravation of moderate to severe arterial hypertension (>160/90 mm Hg) accompanied by manifestations of malignant hypertension (hypertensive grade III or IV retinopathy, pulmonary edema, and/or hypertensive encephalopathy) and elevation of peripheral renin activity to at least twice the upper limit of normal as defined by Traub et al.^43^ “Sicca syndrome” is defined as the presence of ocular and oral dryness, and ocular signs and abnormal salivary gland function tests.

### Nailfold Capillaroscopy

On the basis of the study by Maricq et al,^44^ 2 main capillaroscopic patterns were distinguished: an active pattern characterized by predominant capillary loss, and a slow pattern characterized by the presence of megacapillaries, but no significant capillary loss.

### Immunological Features

Antinuclear antibodies (ANAs) were identified by indirect immunofluorescence (IIF) assay using Hep-2 cell lines or triple tissue cryostat section (liver-stomach-kidney). Anticentromere antibodies (ACAs), anti-Pm-Scl and antibodies to saline extractable nuclear antigens (SSA/Ro, SSB/La, Sm, RNP, and topoisomerase I [ATA]) were determined.

### Causes of Death

Causes of death of SSc patients were classified as SSc-related mortality and unrelated to SSc. SSc-related deaths were defined as deaths related to organ failure directly related to SSc: pulmonary involvement (ILD, PH, or both); heart involvement (ischemic cardiomyopathy, heart failure, or arrhythmias not attributable to other cardiac conditions); renal involvement: SRC and other SSc organ involvement. Unrelated to SSc were all other causes not attributed to SSc organ involvement: infection, cancer, cardiovascular disease, and other causes (a variety of common causes of death not related to SSc).

### Statistical Analysis

This study is a nationwide, retrospective analysis of SSc subsets. Data about death were verified by 2 ways. Patients’ charts were reviewed to determine the last follow-up. Patients were classified as alive if they were followed up until January 2008. For patients deceased in hospital, the cause of death was recorded from charts. In the few cases in whom the date and cause of the patient's death were not available in the hospital's medical records, the information was frequently obtained from their relatives and/or their General Practitioners. If there was no notification, the cause of death was defined as “unknown.” Demographic features and disease characteristics of alive and deceased patients at 2008 were compared. Statistical evaluation was performed using a contingency table test (chi-square test or Fisher exact test when necessary) to identify significant differences. Kaplan–Meier method was used to estimate survival from disease onset (RP in the majority of patients), and Mantel–Haenszel statistic test (log-rank test) to analyze differences in survival among subsets. Univariate comparison between groups was performed for all clinical characteristics and immunological features. Variables that significantly affected survival in the univariate analysis were entered into a multivariate Cox proportional-hazards regression model with both, forward and backward a stepwise selection procedure in order to identify independent risk factors for mortality. Results were consistent in forward and backward procedures. According to a stepwise selection process, variables were entered, or removed, from the regression equation on the basis of a computed significance probability (maximized partial likelihood ratio) and verified by a degree of hands-on modeling. The assumptions for calculating the hazard ratio (HR) were checked by Wald tests. All statistical analyses were performed with SPSS 15.0 for Windows (SPSS, Chicago, IL). The R project was employed for Kaplan–Meier plots including number of patients in risk. A *P* value <0.05 was considered significant.

## RESULTS

### Scleroderma Subsets and Demographic Data of Deceased Patients

By January 2008, 879 consecutive SSc Spanish patients diagnosed according to the modified LeRoy and Medsger^34^ classification from 1970 were recruited on the SSSG database. All patients were Caucasians. Demographic and clinical features are shown in Table 1. Seven hundred forty-nine (85.2%) were women. One hundred thirty-eight (15.7%) deaths were registered overall and 112 (81.2%) of them were women. The mean age at death was 64.1 ± 13.2 years and the estimated mean disease duration from disease onset to death was 7.2 ± 10.7 years. Sixty-nine out of 138 (50%) deceased patients were lcSSc, 63 (45.7%) were dcSSc, and 6 (4.3%) were ssSSc—values significantly different with respect to alive patients at the last follow-up (*P* < 0.0001). Patients who died were older at disease onset and at SSc diagnosis (47.7 ± 15.2 vs 44.4 ± 15.8, and 54.7 ± 14.1 vs 50.6 ± 15.1, respectively).

**TABLE 1:**
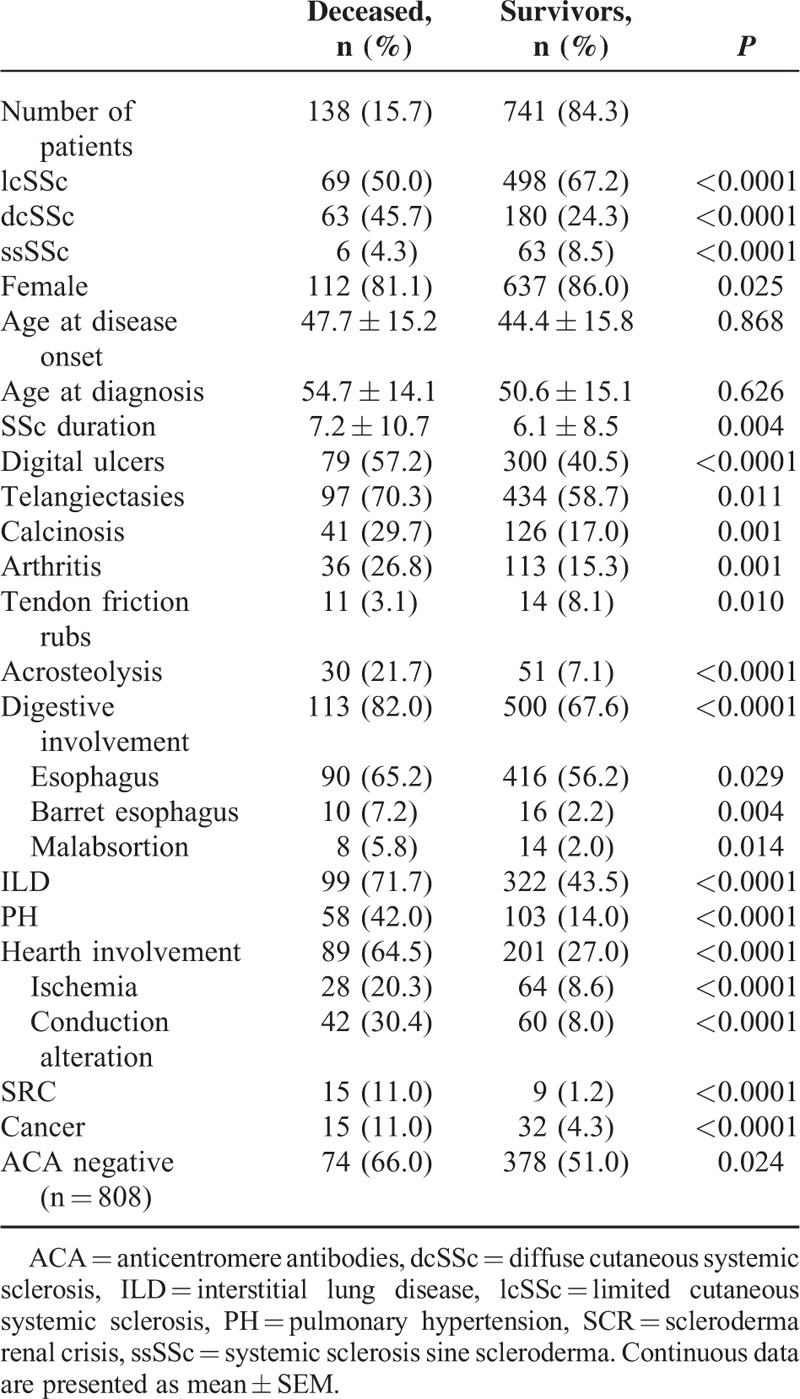
Comparison of Demographic, Clinical, and Immunological Features of Deceased and Survivor Systemic Sclerosis Patients

### Clinical and Immunological Features in Deceased and Alive Patients

Organ involvement and scleroderma manifestations of deceased and alive patients at the assessment time were compared and results are shown in Table 1. In brief, dcSSc, digital ulcers, acro-osteolysis, and organ involvements were more prevalent in patients who died. The cancer incidence in deceased patients was significantly higher than in alive patients. Among the malignancies, breast cancer (15 among 47 malignancies) and lung cancer (9 among 47 malignancies) were clearly most prevalent. The remaining malignancies were distributed as follow: 6 hematologic neoplasm, 5 skin cancer, 4 colon, 3 tiroides, 3 disseminated malignancies of unknown origin, 2 kidney, 2 corpus uteri, and 1 prostate.

### Causes of Death

Seventy-six out of 138 (55%) deceased patients died because of causes directly attributed to SSc, and pulmonary involvement was the main cause. Isolated PH was the most frequent SSc-related cause of death, present in 23 (16.6%) patients, followed by ILD in 18 (13.0%) and PH related to ILD in 17 (12.3%). SRC was a rare cause of death recognized in 12 (8.7%) patients. Sixty-two out of 138 (45%) deceased patients died because of non-SSc–related causes. Cancer was the leading cause in 16 (11.6%) patients, followed by heart failure (not attributable to SSc) in 12 (8.7%) patients. Among the malignancies, lung (4) and breast cancer (3) were most prevalent. When we take into account SSc subtype, the main cause of death was PH in lcSSc patients (17 [24.7%]), ILD in dcSSc patients (13 [20%]), and PH related to ILD in ssSSc patients (2 [33.3%]). The majority of deaths related to SRC occurred in patients with dcSSc. All data are shown in Table 2.

**TABLE 2:**
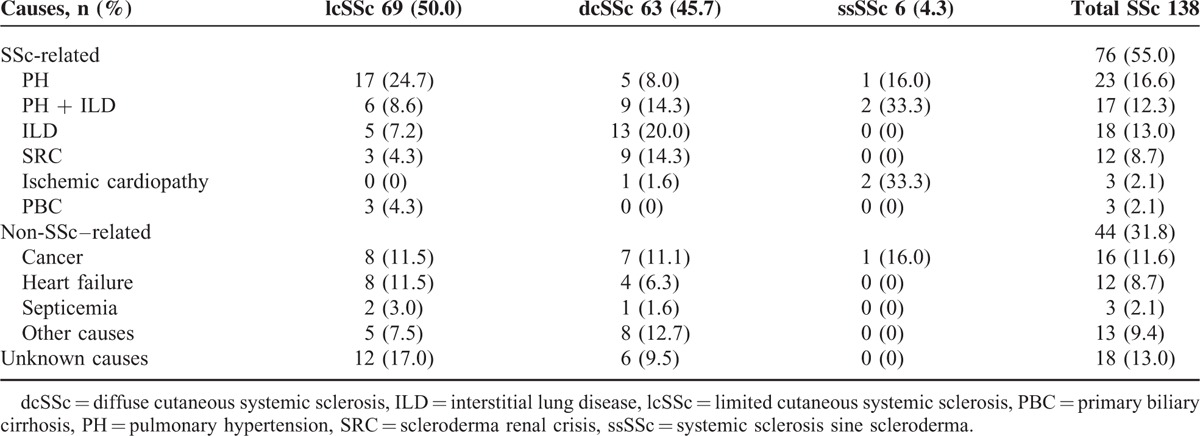
Causes of Death in 138 Spanish Systemic Sclerosis Patients

### Survival Rates

**FIGURE 1:**
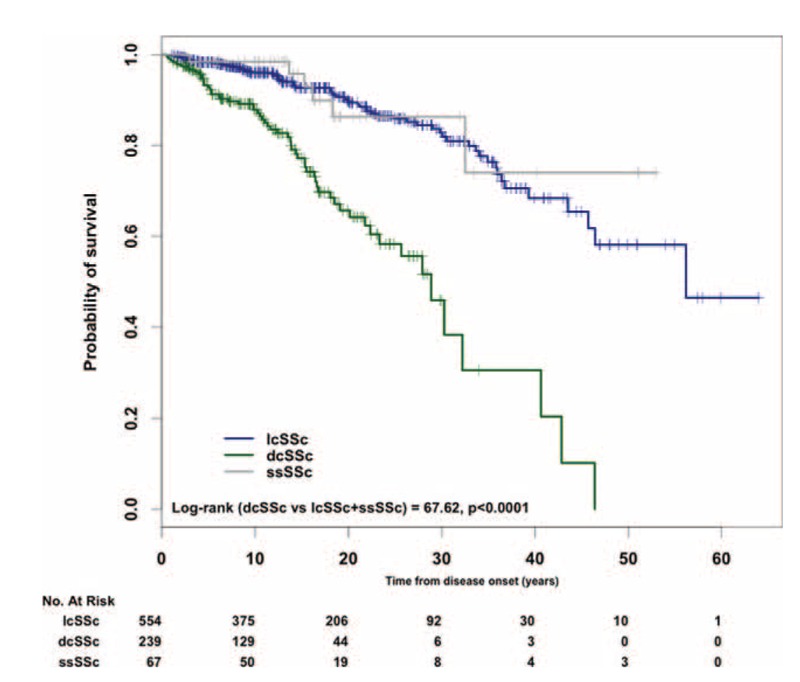
Kaplan–Meier survival curves for systemic sclerosis (SSc) patients from disease onset according to cutaneous subsets of SSc.

Overall, mean survival time was 46.4 ± 4.2 years (95% confidence interval [CI] 38–54) and survival rates were 96%, 93%, 83%, and 73% at 5, 10, 20, and 30 years after the first symptom, respectively. Survival rates for the 3 SSc cutaneous subtypes are shown in Figure 1. Survival rates for lcSSc and ssSSc patients were 96%, 94%, 83%, and 74%; and 98%, 98%, 86%, and 86% at 5, 10, 20, and 30 years, respectively. No statistical differences were found between both groups (*P* *=* 0.755). Survival rates for dcSSc patients were worse than the other subsets—91%, 86%, 64%, and 39% at 5, 10, 20, and 30 years, respectively. Once confirmed that survival rates were similar for lcSSc and ssSSc subsets, both groups were combined in order to compare survival rates of the new largest group (97%, 95%, 85%, and 81% at 5, 10, 20, and 30 years, respectively) with those of the dcSSc subset. The survival rate of the combined group was significantly better than the dcSSc subset (log-rank 67.63, *P* < 0.0001).

**FIGURE 2:**
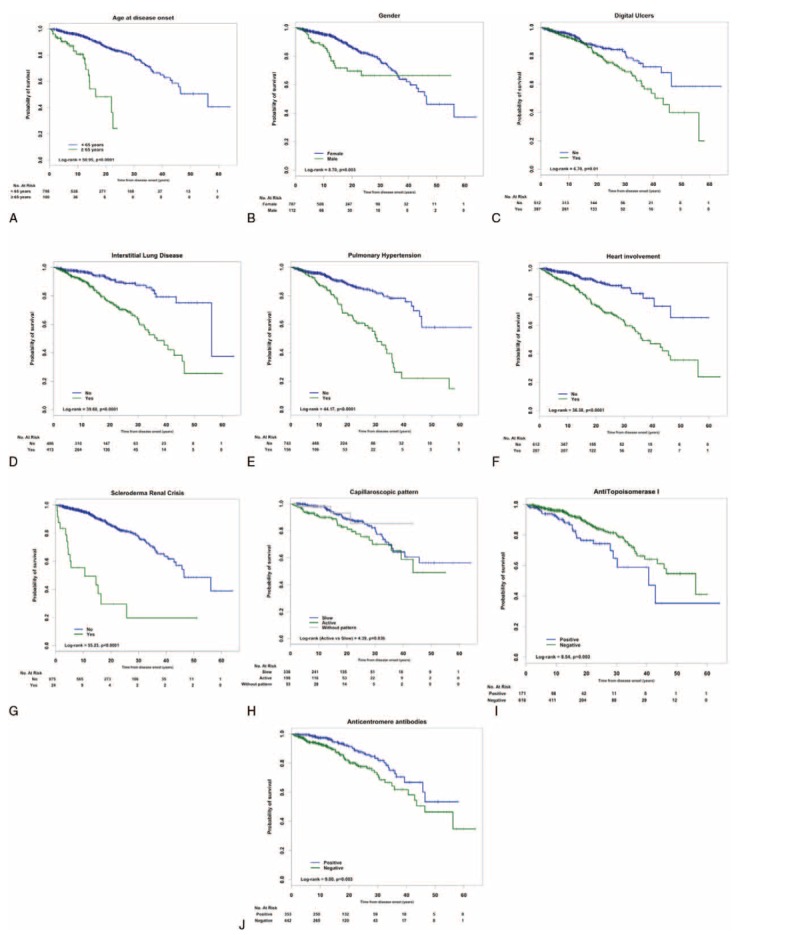
Kaplan–Meier survival curves of patients with systemic sclerosis according to (A) age at disease onset, (B) sex, (C) digital ulcers, (D) interstitial lung disease, (E) pulmonary hypertension, (F) heart involvement, (G) scleroderma renal crisis, (H) capilaroscopic pattern, (I) antitopoisomerase I, and (J) anticentromere antibodies.

The Kaplan–Meier survival curves according to the presence or absence of clinical and immunological features were significantly different on log-rank test, as shown in Figure 2. Pairs of survival curves with significant differences and the survival rates of subgroups at 10, 20, and 30 years after the onset of SSc are shown in Figure 2 and Table 3. Subgroup analysis revealed that the presence of lung, heart, and kidney involvement was associated with worse prognosis.

**TABLE 3:**
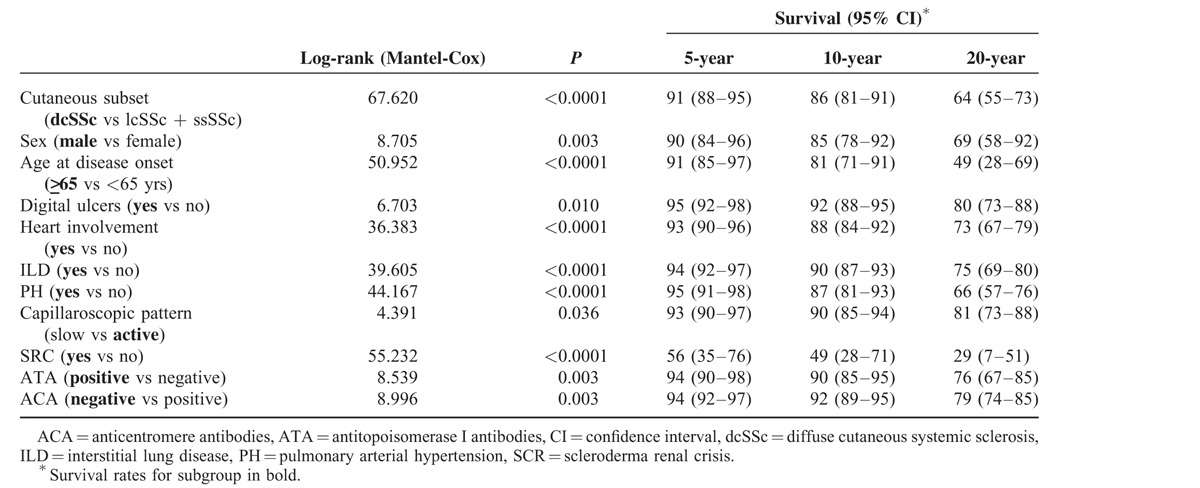
Comparison Between Groups From Log-rank (Mantel Cox) and 5, 10, and 20-Year Survival Rates, Estimated by the Kaplan–Meier Method of Spanish Systemic Sclerosis Patients According to Demographic, Clinical, and Immunological Variables (Crude Analysis)

Of note, dcSSc, male sex, age at first symptom older than 65 years, digital ulcers, ILD, PH, heart involvement, SRC, presence of ATA and absence of ACA, and active capillaroscopic pattern showed significant differences in survival.

### Prognostic Factors

Univariate analysis of all significant parameters (log-rank test) related to mortality showed that dcSSc, male sex, age at first symptom older than 65 years, digital ulcers, ILD, PH, heart involvement, SRC, presence of ATA, and absence of ACA were all associated with diminished survival (Table 4). When multivariate analysis using Cox proportional-hazard model (HR) was performed, older age at disease onset, dcSSc, and the presence of ILD, PH, and/or SRC were confirmed to be independent risk factors for mortality (Table 5).

**TABLE 4:**
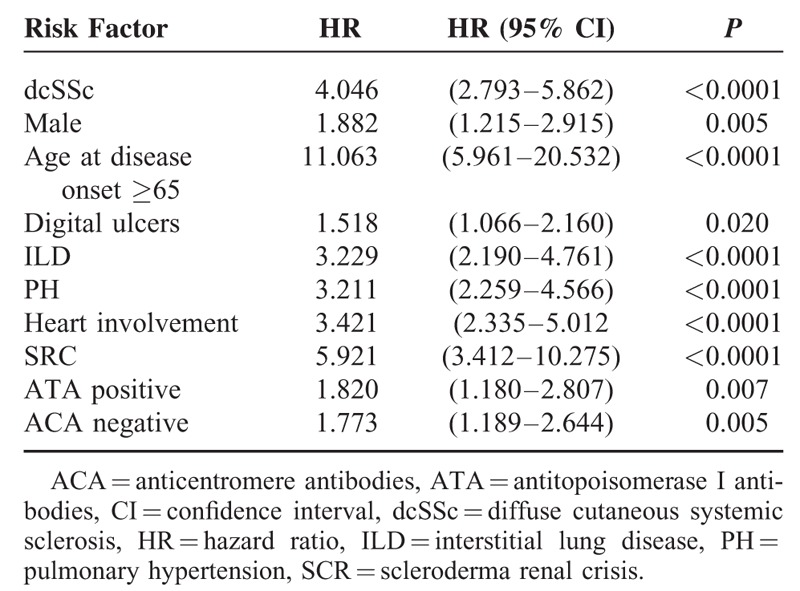
Risk Factors for Reduced Survival in Systemic Sclerosis Patients by Cox Univariate Analysis

**TABLE 5:**
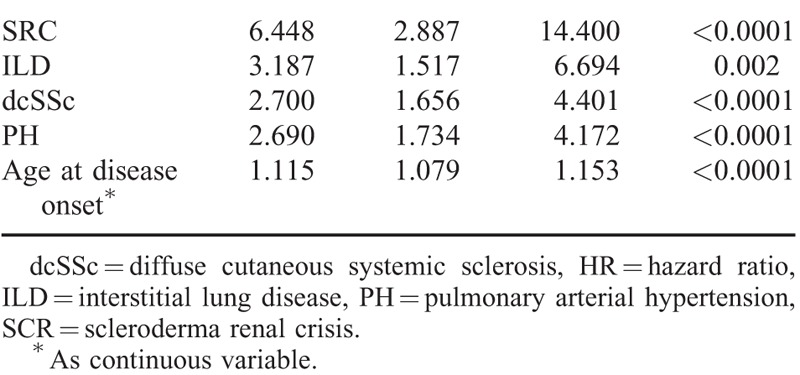
Independent Risk Factors for Reduced Survival of Systemic Sclerosis Patients by Cox Multivariate Regression Modeling

## DISCUSSION

In the present study involving 879 Spanish SSc patients, a high prevalence of disease-related causes of death was demonstrated, and dcSSc, older age at disease onset, ILD, PH, and SRC were identified as independent prognostic factors for mortality. The prevalence of SSc mortality-related causes in our study is similar to those identified in previous registries and meta-analyses.^8,25,31,32,36^ Thus, pulmonary involvement (ILD and/or PH) was the leading cause of death in agreement with previous observations.^8,14,25^ Classically, PH has been considered the main cause of death in patients with lcSSc, but not in patients with dcSSc. However, recent studies showed that this pulmonary vascular complication is a frequent clinical manifestation in all SSc subsets^29,39,45^ and leads to death in many dcSSc patients as well. In the present cohort, 5 and 9 dcSSc patients (22.2%) died because of isolated PH and PH related to ILD, respectively. Since the introduction of the angiotensin-converting enzyme inhibitors (ACEIs), SRC has become a less frequent terminal event in patients with SSc.^8,46^ In the present cohort, SRC accounted for 8.7% of all deaths despite the ACEI treatment. This is a slightly higher incidence than that observed in the Pittsburgh and EUSTAR cohorts,^8,25^ but similar to the reported incidence by the recent meta-analysis.^31,32^ Our data underline that renal involvement plays an important role in SSc mortality yet, and indicates that further efforts are needed to improve management of this complication.

Among non-SSc–related causes of death, malignancies and cardiovascular involvement, in particular, heart failure, were the most frequently identified. In accordance with our results, several studies have suggested an increased incidence of cancer in patients with SSc.^45,47,48^ Specifically, in the Mediterranean area, Airó et al^49^ in a retrospective evaluation of 360 Italian patients with SSc observed a history of malignancies in 10.8% of the patients; in the present study, the prevalence was lower (5.3%), but direct comparison is not possible because of different patient selection and different objective. The malignancies were already identified as a major cause of death in several studies.^2,14,25,31,36,37^

To discriminate SSc from non-SSc–related heart involvement in older patients is sometimes challenging and then we assumed that ischemic cardiomyopathy was SSc-related only in the absence of other vascular risk factors.

In the present cohort, survival rates of SSc patients were better than those described in other studies.^31,39,45,50–54^ Differences may be attributed to methodological and clinical reasons. Firstly, survival was estimated from the first clinical manifestation of SSc, usually RP, according to Domsic and Medsger^1^ recommendations. They stated that RP is the first symptom in the majority of lcSSc patients and in 40% of dcSSc patients. Moreover, the first non-RP symptom occurs at a mean of 5 months after the first symptom attributable to SSc in dcSSc patients.^1^ In agreement with this statement, currently, the majority of studies considered RP as the first manifestation of SSc.^15,16,38–40,55^ Secondly, we used a modified LeRoy et al^34^ classification that allowed recruiting patients with milder clinical SSc variants and reflected the whole spectrum of SSc. It is known that survival of SSc patients has improved over time,^5,7,8,31,33,45,50–54,56^ and older studies usually included a relatively higher percentage of patients with more severe disease. Indeed, in the cohort from Ferri et al; the 10-year survival rate of patients included before 1985 was worse than that of the patients included beyond (60.6% vs 76.8%; *P* < 0.0001).^3^ Probably, this finding is related to the systematic annual screening of the organ-based complications and the improvement of the current organ therapies. Eighty-five percent of patients in our study were diagnosed beyond 1990 and it may explain the high survival rates we identified. Thus, Ferri et al^57^ recently reported an interesting study underlining the evolution of SSc pathomorfosis and prognosis during the time. The authors reviewed the world literature and observed increased median 10-year survival from 54% (patients recruited in 1955–1985) to 83.5% (patients recruited between 1999 and 2011). The authors observed that the worse prognostic factors were less frequent in recently recruited Italian series. Thus, the prevalence of dcSSc, ATA, digital ulcers, and main visceral organ involvement was less commonly detected. In Ferri et al's recent cohort (patients recruited between 2000 and 2011), the cumulative 10th-year survival rate calculated from RP was 95.4%, which is similar to that in the present study (93%).

In our study, survival rates of dcSSc patients were higher compared with those found in some cohorts,^39,45,54^ but our results are in accordance with recent Japanese^15^ and Norwegian^56^ studies.

The present study included a large number of variables potentially influencing survival of SSc patients such as sex, age at disease onset, cutaneous subsets, visceral involvement, and the presence or absence of ACA and ATA. We evaluated the independent predictor factors in terms of HR, and lung involvement (ILD and/or PH), dcSSc, SRC, and age at disease onset older than 65 years were identified as independent factors of lesser survival rate. Although older age at disease onset was found an independent risk factor in many studies,^2,3,5,6,14,18,36^ results should be adjusted according to the matched general population because survival rate usually decreases with aging. Accordingly, Pérez-Bocanegra et al^50^ used standardized mortality ratio (SMR) analysis and concluded that older age at disease onset of SSc had worse survival rate than younger age at disease onset, even though they had lcSSc, which is known as the best prognostic subset. However, when SMR was adjusted to the expected background population mortality rates, mortality was only slightly increased in the subset older than 65 years (SMR 1.2). Hoffman-Vold et al^56^ observed SMR of 2.03.

It is known that dcSSc patients have worse outcome than lcSSc^2,5,14,15,35–37,39,46,51,56^ because of its predisposition to develop internal organ involvement.^29,30,32^ Specifically, lung and renal involvements are more prevalent and severe in the former and may explain the highest mortality evidenced in dcSSc patients. Thus, multivariate analysis identified SRC as a major risk factor for death (HR 6.448) with a low survival rate at 5 years despite an early ACEI therapy in the acute SRC phase.^5,6,16,32^

Interstitial lung disease was the second risk factor for death, with a HR of 3.187. Pulmonary restriction has been described as an independent risk factor in previous studies.^8,25,32,56^ In our study, ILD was a leading cause of death in 25.3%, and it was related to PH in 12.3% of them. Therefore, the development of PH in patients with ILD is associated to worse prognosis.

In this study, PH represented an important independent risk factor of mortality and it was the main cause of death, either isolated (16.6%) or related to ILD (12.3%), in agreement with Hachulla et al,^14^ who observed that PAH was a major risk factor for death with a HR of 7.246 during a 3-year period. These observations underline the increasing impact of pulmonary vascular complication on SSc survival.^14,30–32^

Other factors such as male sex, heart involvement, presence of ATA, and absence of ACA were associated with poor prognosis in the univariate analysis and the survival study. However, we could not demonstrate they were independent risk factors. These results are similar to the EUSTAR study and the Spanish cohort.^25,39^

The main strengths of the present study are the recruitment of a large cohort of SSc patients from 14 hospitals in Spain and the long-term follow-up period, given the relative low prevalence, as well as the homogeneous demographic composition of the sample, because only patients of Spanish origin were included. Nevertheless, some limitations are recognized such as the retrospective design of the survey and the fact that the cause of death was unknown in 18 patients, which represented 13% of deaths. This percentage is similar to that described in a recent meta-analysis (16%)^31^ and in the EUSTAR prospective study (4%).^25^ The SMR was not estimated in the mortality study.

Despite these limitations, we believe that our study represents a real picture of the Spanish SSc patient evolution. Taken together, our data demonstrated a high prevalence of disease-related causes of death being lung involvement. Independent risk factors for mortality were dcSSc, age at the most frequent disease onset, ILD, PH, and SRC. Therefore, improvement on SSc patients’ prognosis is related to advances of therapy for lung involvement.
